# Editorial

**Published:** 1952-04

**Authors:** 


### *Editorial*

### HEALTH CENTRES

It is certainly an advantage that financial stringency has caused delay in the provision of large numbers of health centres. Dr. R. C. Wofinden in this issue describes some of the difficulties encountered in building and organizing the first Centre in Bristol, which is also the first in the country to be built for the purpose.

A Health Centre sounds very nice in theory, but few people have a clear idea how one functions and what work should be done in it. The Bristol plan is based on the simplest form whereby several doctors will carry on orthodox "surgeries" under one roof and share the building with the District Nursing Service and the Local Authority Clinics. It provides for none of the pathological, X-ray or dispensary assistance, which many people consider one of the attractive possibilities in group practice, yet the running costs seem high enough already. It is clear that the experience of running a small number of centres for a year or two in different parts of the country is required before any mass building of centres commences.

### MATERNITY SERVICES

We published in our last number an article by Professor Lennon on Maternity Services in Bristol and this issue includes letters expressing the general-practitioner view on those proposals. It is now learnt that the Bristol Clinical Area Joint Advisory Committee has set up a new Midwifery Committee to advise on the future of the maternity service over the whole of the Clinical Area. Before this new committee was formed, discussions had reached an advanced stage between the groups concerned with a view to the co-ordination of the Maternity Service in the City of Bristol on the lines recommended in the memorandum of the Bristol Local Medical Committee (*Brit. Med. J.*, 195, ii, 126). The main recommendations were that consultants, general practitioners and midwives should combine as a team in all midwifery cases; that every expectant mother whether she had to be confined in hospital or at home would be under the care of her own doctor if she so desired; and that maternity units should be set up with facilities for general practitioners who wish to practise midwifery to do their own cases under hospital conditions.

The new Committee must view these recommendations, which were warmly approved by other Local Medical Committees in the country, in the light of Professor Lennon's proposals and of the partially critical response of the local doctors.

### CANCER PROPAGANDA

Professor Rendle Short has followed up his article on Cancer Propaganda in the *Medical Press* of November 28th, 1951, with a letter in the *British Medical Journal* of February 2nd, 1952.

Briefly, his argument is that if patients would go to their doctor as soon as there were signs or symptoms of cancer, the amount of invalidism could be greatly reduced, and the expectation of life increased. He suggests that a pilot propaganda scheme should be tried in the South West area concentrating on cancer of the breast, uterus and mouth, using the pamphlet or newspaper article rather than the hoarding to tell the patient the need for early diagnosis. Professor Rendle Short does not think that this would lead to cancer phobia, or that the already overworked G.P. would be harassed by a stream of frightened people. It certainly seems worth a trial, and we understand that the Health Committee of Bristol Corporation are likely to give their approval; it is hoped that other Health Authorities will co-operate.

### A NEW M.O.H. FOR TUBERCULOSIS

A weakness of the N.H.S. is that while treatment of tuberculosis is in the hands of the Regional Hospital Board, prevention and after care are maintained by the Local Health Authority. To remedy this defect an assistant Medical Officer of Health is to be appointed in Bristol and will be responsible for the co-ordination of the tuberculosis service. The bewildered G.P. will now have someone to whom to turn when he has patients in the same family, some of whom are under the Hospital Board, and some under the care of the Health Department.

